# Place, Race, and Lapses in Diabetic Retinopathy Care

**DOI:** 10.1001/jamaophthalmol.2024.0974

**Published:** 2024-04-25

**Authors:** Tina Tang, Diep Tran, Dingfen Han, Scott L. Zeger, Deidra C. Crews, Cindy X. Cai

**Affiliations:** 1 Wilmer Eye Institute, Johns Hopkins School of Medicine, Baltimore, Maryland; 2 Department of Medicine, Johns Hopkins School of Medicine, Baltimore, Maryland; 3 Department of Biostatistics, Bloomberg School of Public Health, Johns Hopkins University, Baltimore, Maryland; 4 Department of Biomedical Informatics and Data Science, Johns Hopkins School of Medicine, Johns Hopkins University, Baltimore, Maryland

## Abstract

This cohort study investigates the association of neighborhood-level social determinants of health with lapses in diabetic retinopathy care by race and ethnicity.

Social determinants of health (SDOH) are associated with health outcomes. Individual and neighborhood-level SDOH play critical roles in diabetic retinopathy screening; however, their interaction for eye care use is not well established.^1,2^ Prior studies suggested that neighborhood socioeconomic disadvantage worsened health outcomes in minoritized populations.^3^ We investigated whether the association of neighborhood-level SDOH with risk for lapses in diabetic retinopathy care varied by race and ethnicity.

## Methods

This was a retrospective cohort study of adult patients (aged ≥18 years) with diabetes seen at the Wilmer Eye Institute from 2013 to 2022 for diabetic retinopathy screening or treatment.^4^ The Johns Hopkins Institutional Review Board approved the study and waived the requirement for informed consent because this was secondary research. This study followed the STROBE reporting guideline.

The outcome was a lapse in diabetic retinopathy care over 2 years.^4^ The main exposure was patient neighborhood socioeconomic disadvantage as measured by the 2019 area deprivation index (ADI).^5^ ADI was divided into quartiles (Qs), with Q4 indicating the most socioeconomic disadvantage. Baseline characteristics were extracted from the electronic health record, including race and ethnicity, other demographic data, clinic, residential distance from clinic, severity of diabetic retinopathy, other retinal disorders, and glaucoma.^4,5^ Multivariable logistic regression models were constructed to assess the association between ADI quartile and lapses in care, controlling for baseline characteristics. The significance of an interaction term between race and ethnicity and ADI quartile was assessed using the likelihood ratio test. Pairwise comparisons were made using contrast coefficients adjusted with Bonferroni correction. The predicted probability of lapses in diabetic retinopathy care was calculated (eMethods in Supplement 1).

## Results

Among 36 497 patients (Table), 63% had a lapse in care, including 60% in ADI Q1, 62% in Q2, 66% in Q3, and 68% in Q4. There was an interaction between ADI and race and ethnicity (*P* = .005) (Figure). Non-Hispanic White patients from more socioeconomic disadvantaged neighborhoods had increased odds of lapses in care (Figure). In pairwise comparisons, non-Hispanic Black and Hispanic patients and patients with other race or ethnicity from higher ADI quartiles had higher odds of lapses in care compared with non-Hispanic White patients in Q1 and Q2. Non-Hispanic Black patients from Q1 had lower odds of lapses in care compared with non-Hispanic White patients from Q4.

**Table.** Baseline Characteristics of Patients

Characteristic	Patients, No. (%)^a^				
	Total (N = 36 497)	ADI Q1 (n = 13 335)	ADI Q2 (n = 12 330)	ADI Q3 (n = 6025)	ADI Q4 (n = 4511)
Age, y					
≤20	98 (0.3)	35 (0.3)	36 (0.3)	12 (0.2)	14 (0.3)
>20 to 45	4357 (11.9)	1198 (9.0)	1550 (12.6)	877 (14.6)	695 (15.4)
>45 to 65	17 429 (47.8)	5831 (43.7)	5861 (47.5)	3054 (50.7)	2529 (56.1)
>65	14 613 (40.0)	6271 (47.0)	4883 (39.6)	2082 (34.6)	1273 (28.2)
Sex					
Female	19 155 (52.5)	6217 (46.6)	6462 (52.4)	3497 (58.0)	2806 (62.2)
Male	17 342 (47.5)	7118 (53.4)	5868 (47.6)	2528 (42.0)	1705 (37.8)
Race and ethnicity^b^					
Hispanic	1759 (4.8)	508 (3.8)	643 (5.2)	353 (5.9)	241 (5.3)
Non-Hispanic Black	13 570 (37.2)	2447 (18.4)	4116 (33.4)	3323 (55.2)	3499 (77.6)
Non-Hispanic White	17 153 (47.0)	8284 (62.1)	6317 (51.2)	1970 (32.7)	510 (11.3)
Other	4015 (11.0)	2096 (15.7)	1254 (10.2)	379 (6.3)	261 (5.8)
Insurance					
Private	13 753 (38.5)	5910 (44.9)	4853 (40.4)	1870 (31.9)	1035 (23.7)
Medicare	14 458 (40.5)	5365 (40.8)	4774 (39.7)	2444 (41.7)	1756 (40.3)
Medicaid	3858 (10.8)	542 (4.1)	1046 (8.7)	1015 (17.3)	1194 (27.4)
Other	2787 (7.8)	1238 (9.4)	1080 (9.0)	280 (4.8)	179 (4.1)
None	837 (2.3)	95 (0.7)	273 (2.3)	254 (4.3)	197 (4.5)
Clinic					
Retina clinic	3594 (9.9)	1231 (9.2)	1141 (9.3)	650 (10.8)	533 (11.8)
Resident clinic	5304 (14.5)	469 (3.5)	1318 (10.7)	1446 (24.0)	1965 (43.6)
Other	27 599 (75.6)	11 635 (87.3)	9871 (80.1)	3929 (65.2)	2013 (44.6)
DR severity					
No DR	28 981 (79.4)	10 840 (81.3)	9834 (79.8)	4653 (77.2)	3432 (76.1)
NPDR	5478 (15.0)	1836 (13.8)	1803 (14.6)	1013 (16.8)	779 (17.3)
PDR	2038 (5.6)	659 (4.9)	693 (5.6)	359 (6.0)	300 (6.7)
Other retinal disorders					
Absent	34 988 (95.9)	12 645 (94.8)	11 819 (95.9)	5831 (96.8)	4405 (97.7)
Present	1509 (4.1)	690 (5.2)	511 (4.1)	194 (3.2)	106 (2.4)
Glaucoma					
Absent	30 887 (84.6)	11 476 (86.1)	10 472 (84.9)	5011 (83.2)	3672 (81.4)
Present	5610 (15.4)	1859 (13.9)	1858 (15.1)	1014 (16.8)	839 (18.6)
Distance from nearest ophthalmology clinic, mean (SD), miles	12 (74.4)	12 (85.8)	13 (67.5)	12 (65.9)	10 (66.6)
ADI score, mean (SD)	40 (25.5)	15 (6.3)	37 (7.1)	62 (7.4)	88 (7.0)

Abbreviations: ADI, area deprivation index; DR, diabetic retinopathy; NPDR, nonproliferative diabetic retinopathy; PDR, proliferative diabetic retinopathy; Q, quartile.

^a^ Study population consisted of patients with diabetes seen for DR screening or treatment stratified by ADI quartile. Q1 has the least and Q4 has the most neighborhood socioeconomic disadvantage.

^b^ Race and ethnicity were recorded separately in the electronic health record and combined for this study. Patients were categorized as Hispanic if either self-reported race or ethnicity was Hispanic. Patients were categorized as non-Hispanic White if race was White or Caucasian and ethnicity was not Hispanic or Latino. Patients were categorized as non-Hispanic Black if race was Black or African American and ethnicity was not Hispanic or Latino. Other race and ethnicity included Asian, American Indian or Alaska Native, Native Hawaiian or Other Pacific Islander, other, unknown, choose not to disclose, unable to obtain, and 2 or more races.

**Figure.**
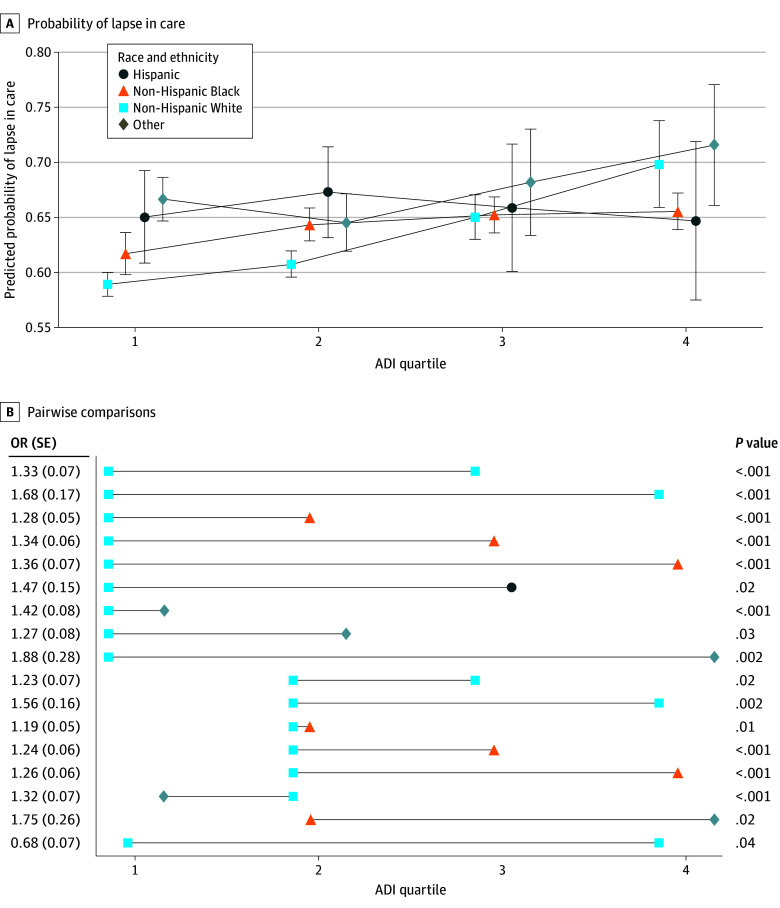
Probability of Lapse in Care and Pairwise Comparisons A, The predicted probability (with 95% CI) of having a lapse in diabetic retinopathy care is given by area deprivation index (ADI) quartile (with quartile 4 representing the most and quartile 1 the least socioeconomic disadvantage) and race and ethnicity. B, Results from the pairwise comparison are displayed, including odds ratio (OR) and *P* value.

## Discussion

Place clearly matters; areas with socioeconomic disadvantage often have concentrated poverty, poor walkability, limited access to public transportation, few health care facilities, and other factors that can make eye care follow-up challenging.^2,3^ This is likely why this cohort study found that non-Hispanic White patients from more socioeconomically disadvantaged neighborhoods were more likely to have lapses in diabetic retinopathy care compared with those from less disadvantaged neighborhoods. Additionally, non-Hispanic Black and Hispanic patients and those with other race or ethnicity from nearly all neighborhoods were more likely to have lapses in care compared with non-Hispanic White patients from the least socioeconomically disadvantaged neighborhoods. For racial and ethnic minority groups, the impacts of structural racism and its downstream effects on the maldistribution of individual-level resources may compound the adverse outcomes associated with neighborhood socioeconomic disadvantage.^6^ Our finding that non-Hispanic Black patients from Q1 were less likely to have lapses in care compared with non-Hispanic White patients from Q4 suggests that living in a neighborhood with less socioeconomic disadvantage may be associated with reductions in adverse effects of structural racism on eye care use among non-Hispanic Black people. Limitations include the study’s retrospective nature and limited generalizability given that it was conducted at a single academic institution. Both place and individual-level factors are associated with diabetic retinopathy care. Given that health and health behaviors represent a complex interplay among individual, community, and societal factors, future studies of diabetic retinopathy care should consider individual and neighborhood-level SDOH and their interactions toward the goal of informing public policies to eliminate disparities in vision health.

## Notes

Supplement 1. **eMethods.** Statistical Methods

Supplement 2.
Data Sharing Statement

## References

[1] Taccheri C, Jordan J, Tran D, . The impact of social determinants of health on eye care utilization in a national sample of people with diabetes. Ophthalmology. 2023;130(10):1037-1045. doi:10.1016/j.ophtha.2023.06.007 37329902 PMC10528242

[2] Yusuf R, Chen EM, Nwanyanwu K, Richards B. Neighborhood deprivation and adherence to initial diabetic retinopathy screening. Ophthalmol Retina. 2020;4(5):550-552. doi:10.1016/j.oret.2020.01.016 32139294 PMC8281953

[3] Gaskin DJ, Thorpe RJ Jr, McGinty EE, . Disparities in diabetes: the nexus of race, poverty, and place. Am J Public Health. 2014;104(11):2147-2155. doi:10.2105/AJPH.2013.301420 24228660 PMC4021012

[4] Cai CX, Tran D, Tang T, . Health disparities in lapses in diabetic retinopathy care. Ophthalmol Sci. 2023;3(3):100295. doi:10.1016/j.xops.2023.100295 37063252 PMC10090804

[5] Cai CX, Klawe J, Ahmad S, . Geographic variations in gender differences in cataract surgery volume among a national cohort of ophthalmologists. J Cataract Refract Surg. 2022;48(9):1023-1030. doi:10.1097/j.jcrs.0000000000000938 35318293 PMC9415203

[6] Elam AR, Tseng VL, Rodriguez TM, Mike EV, Warren AK, Coleman AL; American Academy of Ophthalmology Taskforce on Disparities in Eye Care. Disparities in Vision Health and Eye Care. Ophthalmology. 2022;129(10):e89-e113. doi:10.1016/j.ophtha.2022.07.010 36058735 PMC10109525

