# Supplementary material for: Place, Race, and Lapses in Diabetic Retinopathy Care
Source: JAMA Ophthalmol. 2024 Apr 25;142(6):581–3. doi: 10.1001/jamaophthalmol.2024.0974 (PMC11046402; doi:10.1001/jamaophthalmol.2024.0974)
Supplement: Supplement 1. — eMethods. Statistical Methods [file jamaophthalmol-e240974-s001.pdf]

# Supplemental Online Content

Tang T, Tran D, Han D, Zeger SL, Crews DC, Cai CX. Place, race, and lapses in diabetic retinopathy care. *JAMA Ophthalmol*. Published online April 25, 2024.
doi:10.1001/jamaophthalmol.2024.0974

**eMethods.** Statistical Methods

This supplemental material has been provided by the authors to give readers additional information about their work.

© 2024 Tang T et al. *JAMA Network Open.*

**eMethods.** Statistical Methods

Statistical significance was set at $P < .05$. Analyses were performed using Stata statistical software version 18 (StataCorp) and Python programming language version 3.8.5 (Python Software Foundation).

© 2024 Tang T et al. *JAMA Network Open.*
