# Supplementary material for: Place, Race, and Lapses in Diabetic Retinopathy Care
Source: JAMA Ophthalmol. 2024 Apr 25;142(6):581–3. doi: 10.1001/jamaophthalmol.2024.0974 (PMC11046402; doi:10.1001/jamaophthalmol.2024.0974)
Supplement: Supplement 2. — Data Sharing Statement [file jamaophthalmol-e240974-s002.pdf]

Data Sharing Statement

Tang. Place, Race, and Lapses in Diabetic Retinopathy Care. *JAMA Ophthalmol*. Published April 25, 2024. doi:110.1001/jamaophthalmol.2024.0974

Data
Data available: No
